# Intraovarian platelet-rich plasma injection in poor responders

**DOI:** 10.5935/1518-0557.20240031

**Published:** 2024

**Authors:** Luciana María Devenutto, Gastón Rey Valzacchi, Marianela Ercolano, Orlando Etchegoyen

**Affiliations:** 1 Procrearte, Buenos Aires, Argentina

**Keywords:** ovarian rejuvenation, oocyte activation, ovarian function, oocyte donation, IVF, pregnancy

## Abstract

**Objective:**

To evaluate if it possible to improve ovarian reserve parameters and oocyte retrieval in poor responders who undergo intraovarian injection of platelet-rich plasma (PRP).

**Methods:**

Prospective cohort study. We included 148 poor responders who underwent PRP injection between October 2021 and December 2022 in our institution, comparing pre and post PRP ovarian function. In addition, the IVF outcomes of a subgroup of patients was studied after the intervention in contrast with the previous treatment.

**Results:**

An improvement in ovarian reserve was observed in relation to previous values: FSH (13.57 vs. 11.32, *p*=0.11), AMH (0.39 vs. 0.48, *p*=0.06), antral follicle count (3.98 *vs*. 5.75, *p*<0.001); as well as a higher number of oocytes retrieved (2.63 *vs*. 3.65, *p*=0.01) and produced embryos (1.64 *vs*. 2.22, *p*=0.03); without a great impact on pregnancy rates.

**Conclusions:**

Although experimental, intraovarian PRP could restore ovarian function and be postulated as an alternative to oocyte donation in patients with low ovarian reserve who do not accept this treatment. There is a lack of randomized controlled trials to support these findings.

## INTRODUCTION

Ovarian failure due to ovarian aging in women of advanced reproductive age is one of the main causes of infertility around the world (American College of Obstetricians and Gynecologists Committee on Gynecologic Practice and Practice Committee, 2014). It involves a decrease in both quantity and quality of oocytes, with the consequent compromise in assisted reproduction treatment outcomes, in terms of low fertilization and blastulation rates and high aneuploidy rates (Kasapoğlu & Seli, 2020). There is also a reduction in ovarian volume, with increased fibrosis and loss of ovarian structure (Nicosia, 1987).

The so-called “poor responders”, defined by a combination of decreased ovarian reserve parameters and low oocyte retrieval after ovarian stimulation, have accelerated ovarian aging (Cakiroglu *et al*., 2022). This population represents 9-24% of patients undergoing in vitro fertilization (IVF), which means that up to one in four patients will have a poor reproductive prognosis (Galatis *et al*., 2022; Vaiarelli *et al*., 2018).

Recently, although different approaches have been introduced to improve this prognosis, there is still a lack of effective strategies available. Within an experimental framework, in order to promote follicle activation and increase the number of retrieved oocytes, ovarian fragmentation with or without in vitro activation (drug-free IVA) and subsequent autologous transplantation (Devenutto *et al*., 2020); as well as autologous ovarian stem cell transplantation (Herraiz *et al*., 2018), have been described. These techniques are both invasive and not yet included in randomized trials.

Interest in this subject has arisen from the observation of residual follicles on ovarian cortex biopsies from patients with primary ovarian insufficiency (POI) (van Kasteren & Schoemaker, 1999); as well as the possibility of reactivating “quiescent” or “dormant” follicles which resulted in pregnancies through in vitro ovarian activation (IVA technique) by the incubation with PTEN (tensin-homologous phosphatase) and PI3K (phosphatidylinositol 3-kinase) inhibitors, and AKT (serine/threonine protein kinase 1) stimulants (Li *et al*., 2010).

Along the same lines, a much less invasive procedure being researched, is the intraovarian injection of platelet-rich plasma (PRP). This is a concentrate derived from centrifuged whole blood that contains up to seven times more platelets than those in circulating plasma, and its regenerative properties are due to its high concentrations of growth factors such as: TGF-β (transforming growth factor-β), IGF-1 and IGF-2 (insulin-like growth factors 1 and 2), VEGF (vascular endothelial growth factor), EGF (epidermal growth factor), bFGF (basic fibroblast growth factor) and HGF (hepatocyte growth factor) (Melo *et al*., 2020). Several of these factors promote tissue healing and regeneration by inducing chemotaxis, cell migration and differentiation. In addition, they contribute to angiogenesis and inflammatory changes that play a key role in tissue repair and regeneration (Dhurat & Sukesh, 2014; Everts *et al*., 2020).

It has been suggested that PRP has the potential to delay follicle atresia and oocyte degeneration (Melo *et al*., 2020), as well as to promote the development of primordial and primary follicles up to the pre-antral stage (Hosseini *et al*., 2017). Another benefit is that, due to its autologous nature, it has no risk of transmissible diseases and immune rejection (Park *et al*., 2020).

Numerous studies demonstrate a restoration of ovarian function in women with diminished ovarian reserve 2 or 3 months after PRP injection (Cakiroglu *et al*., 2022; Melo *et al*., 2020; Sabouni *et al*., 2021; Sills *et al*., 2018; Tülek & Kahraman, 2022); as well as an improvement of the ovarian reserve parameters [decrease in FSH (Melo *et al*., 2020; Sills *et al*., 2018), increase in AMH (Melo *et al*., 2020; Sabouni *et al*., 2021; Sfakianoudis *et al*., 2020; Pacu *et al*., 2021) and an increase in antral follicle count (Cakiroglu *et al*., 2022; Melo *et al*., 2020; Sfakianoudis *et al*., 2020; Pacu *et al*., 2021)]. Furthermore, an increase in the number of retrieval oocytes and produced embryos after ovarian stimulation has also been reported. There have also been numerous pregnancies and live births after the application of this technique (Tülek & Kahraman, 2022).

The aim of the present study was to describe ovarian reserve parameters and IVF outcomes in a cohort of 148 poor responders treated with intraovarian injection of autologous PRP. We hypothesized that intraovarian injection of PRP may improve ovarian reserve parameters and oocyte retrieval in poor responders undergoing an assisted reproductive treatment.

## MATERIALS AND METHODS

### Study design and patient selection

Prospective observational cohort study of ovarian reserve parameters and IVF outcomes in poor responders after intraovarian injection of autologous PRP. This study was conducted at the Reproductive Medicine Center “Procrearte”, Buenos Aires, from October 2021 to December 2022.

148 patients under 45 years old were included, all of whom had previously undergone at least one assisted fertilization treatment with a recovery of less than 5 oocytes and/or a low ovarian reserve profile (Poseidon 1, 2, 3 and 4). Low ovarian reserve was defined as: AMH <1 ng/ml and/or early follicular phase antral follicle count <5.

Patients with oncological disease, history of chemoor radiotherapy, severe cardiac disease, ovarian and/or deep endometriosis, polycystic ovarian disease, active sexually transmitted disease, multiple previous pelvic surgeries, platelet function disorder, moderate or severe thrombocytopenia, coagulopathy and anticoagulant treatment were excluded.

Ovarian function was performed in each patient through hormonal assays (FSH, LH, oestradiol and AMH) and total antral follicle count, in order to compare the previous values (within 6 months before the PRP injection) with those at 3 months post-procedure. In addition, for patients who underwent ART, we compared the number of oocytes retrieved, the number of MII oocytes, the fertilization rate, the number of 2 pronuclear and total produced embryos after PRP injection with respect to the last treatment preceding this therapy. Spontaneous and post-ART pregnancies were recorded up to the time of conclusion of this study.

All participants signed an informed consent form for the procedure, which explained the experimental approach, as well as the possible associated risks. Patients who chose to participate returned a signed copy of the form to the clinic.

### Procedures

For each patient, 60 ml of blood was obtained under sterile conditions from the median antebrachial vein two hours before the intraovarian injection. All patients were instructed not to take aspirin for 7 days prior to the procedure and fasted for a minimum period of 6 hours.

The blood was placed in two sterile 50 ml tubes with 7.5 ml ACD-A (anticoagulant solution dextrose citrate) (ratio 1 vol ACD-A: 4 vol blood). Double centrifugation was performed at 2000 and 2500 rpm, for 6 and 10 minutes respectively. The platelet concentrate was suspended and homogenized in 7.5 ml of autologous plasma at a concentration of 1.5 x 10^6^ /ul. In addition, 2.5 ml of physiological solution was added since dilution increases the regenerative and neovascularization effect by diluting growth factors that inhibit this function. The suspension was then placed in a refrigerator at 4°C for 30 minutes and activated with 22-25 mL CaCl_2_ at 10%, i.e. for 10 mL, 1 mL of CaCl_2_ was added. PRP preparation was performed in a restricted access area, under aseptic conditions and using a laminar flow hood.

Before the intervention, an assessment of the clinical status of the patients was carried out with a complete blood hemogram, coagulogram, monitoring of renal function, electrocardiogram and serologies (VIH, hepatitis B, hepatitis C, syphilis). In addition, ovarian reserve parameters were evaluated with hormonal profile (FSH, LH, estradiol and AMH) and transvaginal ultrasound with antral follicle count between the second and fourth day of the menstrual cycle.

PRP injection was performed in all cases at least 2 months after the last failed fertility treatment, at the follicular phase (day 7 to 10 of the menstrual cycle). The patient was prepared according to our institution’s ovarian aspiration puncture protocol, in a dorsal lithotomy under local anesthesia or neurolept anesthesia. Firstly, both ovaries were visualized by transvaginal ultrasound, accessing the central portion of the ovaries through a 17 gauge 30 cm length Cook® single lumen needle. Subsequently, gradual infusion was performed in the subcortical and stromal area, using 3 mL of activated PRP per ovary, through a 5 ml syringe connected to the silicone plug of the needle. Although the ovaries of elderly maternal age and poor responders may be small and fibrotic, injection was achieved by creating new planes through distension and injection at multiple sites. The maximum time taken was 20 minutes.

After the procedure, patients were taken to the recovery room and discharged the same day after an initial examination period of 30-40 minutes. Antibiotic prophylaxis was indicated according to our institution’s follicular ovarian puncture protocol. After the operation, the pelvis was thoroughly examined by ultrasound, in order to check total vascular integrity. The supine position was recommended for 15 minutes after the infusion.

During the third month after injection, ovarian function was monitored by hormone profile (FSH, LH, estradiol and AMH) and antral follicle count by transvaginal ultrasound, between the 2nd and 5th days of the menstrual cycle.

According to the patient’s response (at least three antral follicles visualized by transvaginal ultrasound) and the couple’s preferences, ART was initiated in the third menstrual cycle after the procedure, using a protocol with gonadotropin-releasing hormone (GnRH) antagonists and 300 IU of gonadotropins (follitropin alfa and/or human menopausal gonadotropin) from day 2 of the cycle. We prescribed a GnRH antagonist once the follicle diameter reached 14 mm and/or estradiol levels were at 300-400 pg/ml. Ovulation was triggered with recombinant human chorionic gonadotropin when follicles reached 18 mm. Oocyte retrieval was performed 36 hours after discharge and then oocytes were inseminated by intracytoplasmic sperm injection (ICSI).

The embryo transfer was performed in the operating room under transabdominal ultrasound guidance following the usual protocols of the procedure, between days 3 to 6 post puncture, according to medical criteria. Luteal phase support consisted of vaginal micronized progesterone (600 mg daily), until quantitative human chorionic gonadotropin (hCG) tests were obtained fourteen days after embryo transfer.

The cryopreserved embryo transfer cycles were all artificial, and included the indication of oral oestrogens and vaginal progesterone.

### Statistical analysis

Quantitative variables were described by mean and standard deviation. Differences in quantitative variables between groups were compared by t-test and qualitative variables by chi-square test. Statistically significant differences were considered for those probabilities less than 0.05. Statistical analysis was performed with Epi Info 7.2.5.0 software.

## RESULTS

The 148 patients included in the study had an average age of 39.61 years (33-44). At the time of the procedure, they had undergone 1 to 6 ovarian stimulations for ART (mean 1.63). Regarding ovarian function, an improvement in AMH, FSH and antral follicle count was obtained after PRP injection. This last parameter was statistically significant (Table 1).

**Table 1:** Ovarian reserve before and after PRP treatment.

	Before PRP	After PRP	*p*
FSH (mUI/mL)	13.57±8.94	11.32±7.45	0.11
LH (mUI/mL)	6.61±3.09	7.36±6.51	0.41
Estradiol (pg/mL)	77.17±103.59	78.26±76.36	0.93
AMH (ng/mL)	0.39±0.33	0.48±0.39	0.06
Antral follicle count	3.98±2.29	5.75±2.82	**<0.001**

A subanalysis of the outcomes was performed according to different age ranges categorized as group 1 (under 40 years), group 2 (40-42 years) and group 3 (over 42 years). The significant improvement in antral follicle count was only persistent in groups 1 (4.38 *vs*. 6.04; *p*=0.01) and 2 (3.5 *vs*. 6; *p*=0.001), but not in those over 42 years of age (3.87 *vs*. 4.94; *p*=0.11). The corresponding AMH values for each group were 0.49 *vs*. 0.52, *p*=0.17; 0.44 *vs*. 0.47, *p*=0.64; and 0.32 *vs*. 0.42, *p*=0.14; respectively.

At the time of finalization of this study 97 ARTs had been performed, all of which were indicated at the third cycle post intra-ovarian PRP injection. Of the initial 148 patients, there was a loss in follow-up of 9.45% (14 cases); and 6 spontaneous pregnancies (4.05%) were noticed between 2 and 8 months post therapy, 5 of which were ongoing pregnancies and 1 culminated in miscarriage.

Of the remaining 128 patients, 31 finally decided not to undergo treatment with their own oocytes. Oocyte vitrification was performed for maternity postponement in 13 of the remaining 97 cases. Thus, of the initial sample, 84 patients underwent ART for reproductive purposes. We registered 10 post-IVF pregnancies (10/84=11.91%), of which 7 were ongoing (1 twin), 1 live birth, 1 ectopic pregnancy and 1 miscarriage (at week 9 of gestational age).

There were 10 cases of ovarian stimulation failure (11.91%), which did not undergo follicular aspiration puncture. There were 7 patients (8.33%) without oocyte retrieval (9.52%), 3 with immature oocytes (3.57%), 6 cases of fertilization failure (7.14%), 7 arrested embryos (8.33%), and 12 patients (14.28%) who underwent preimplantation genetic testing for aneuploidy (PGT-A) resulting in aneuploid (10 cases) or arrested embryos (2 cases).

Therefore, out of the total sample, only 39 patients were suitable for embryo transfer. Six had not yet undergone it. One patient performed a fresh embryo transfer, followed by a cryopreserved one. In 64.71% of the cases a single embryo was transferred, while in 35.29%, 2 embryos were transferred. A total of 67.65% of the transfers corresponded to embryos at 120 or 144 hours of development.

The pregnancy rate in the group of patients who transferred at least one embryo was 29.41% (10/34), consisting of 4 positive after embryo transfers (4/19) and 6 positive after cryopreserved ones (6/15). Currently, there are 7 ongoing pregnancies, 2 live births and 1 ectopic pregnancy.

A subgroup of 20 patients underwent pre-implantation genetic testing for aneuploidy (PGT-A) and had 26 suitable embryos on day 5 of development. Nine of them were euploid (34.6%) and 17 were aneuploid (65.35%).

An analysis of the 97 ART cases was performed, comparing the outcomes after PRP injection with the last treatment prior to PRP in each case. The average age of these patients was 39.28 years (33-44). In this group, a significant improvement in ovarian reserve was observed with respect to previous values (assessed by AMH and antral follicle count); as well as better results in number of oocytes retrieved, number of mature oocytes (MII), number of 2 pronuclei and evolved embryos; compared to the cycle prior to the therapy. There was no difference in the fertilization rate between the two groups (Table 2).

**Table 2:** ART outcomes.

	Before PRP	After PRP	*p*
FSH (mUI/mL)	13.71±9.11	10.64±4.81	0.06
LH (mUI/mL)	6.41±3.21	6.17±3.21	0.75
Estradiol (pg/mL)	60.68±58.02	49.72±21.86	0.25
AMH (ng/mL)	0.46±0.29	0.62±0.36	**0.03**
Antral follicle count	4.5±2.09	6.15±2.58	**<0.001**
Number of retrieved oocytes	2.63±2.42	3.65±3.17	**0.01**
MII oocytes	2.17±2.07	3.09±3.06	**0.01**
Number of inseminated oocytes	2.34±2.27	3.46±3.13	**0.01**
Number of 2 pronuclei embryos	1.62±1.81	2.31±2.08	**0.02**
Fertilization rate	(1.62/2.34) 69.23%	(2.31/3.46) 66.76%	0.77
Number of abnormal fertilized embryos	0.16±0.42	0.25±0.65	0.41
Number of produced embryos	1.64±1.84	2.22±2.07	**0.03**
Number of evolved embryos	0.78±0.89	1.46±1.27	**0.01**

No complications or adverse effects were recorded in the cases performed during the period of this study.

Descriptively, patients who achieved pregnancy had a mean age of 38.18 years (35-43), AMH of 0.56 ng/ml (0.02-0.71) and antral follicle count of 3.33 (0-6) prior to intraovarian PRP therapy.

## DISCUSSION

Intraovarian PRP injection was recently introduced as an alternative to egg donation in patients with poor reproductive prognosis (Sills *et al*., 2020).

Regarding its mechanism of action, two hypotheses are proposed: the more controversial one introduces the concept of neo-oogenesis, suggesting the presence of ovarian stem cells as a source of oocytes in adult ovaries (Seckin *et al*., 2022). Numerous studies have shown that it is possible to obtain mitotically active germ cells from healthy adult ovarian tissue in mice and humans (Zou *et al*., 2009; White *et al*., 2012); however, there is no evidence that spontaneous stem cell reactivation occurs naturally in the adult human ovary. Another possible explanation is that PRP could activate the development and maturation of “dormant” or quiescent primordial follicles, increasing the pool of ovulatory follicles (Seckin *et al*., 2022).

PRP-derived growth factors include multiple regulatory proteins that bind to cell membrane receptors and direct important chemical messages. Through this interaction, they trigger interand intracellular signalling mechanisms that direct growth, proliferation and differentiation of cells (Sills *et al*., 2020). Unlike hormones, PRP growth factors act only in the proximity of their release site, playing an important role in the restoration of the ovarian niche, mainly by promoting physiological processes of angiogenesis, proliferation and growth, apoptosis, control of inflammation and cell migration (Alves & Grimalt, 2018; Krüger *et al*., 2013; Ozcan *et al*., 2020; Foster *et al*., 2009).

In the last decade, numerous studies have reported that injecting plasma directly into the ovary increases folliculogenesis and restores ovarian function and hormonal profile, with a consequent improvement in oocyte retrieval in patients undergoing ART (Sfakianoudis *et al*., 2020). The first results were reported by Pantos et al., who demonstrated the possibility of restoration of ovarian function in a cohort of eight perimenopausal women undergoing IVF, with successful oocyte retrieval (Pantos *et al*., 2016).

In this study we investigated whether intraovarian injection of PRP improves ovarian reserve and IVF outcomes in poor responders. The decision to initiate ovarian stimulation protocol 3 cycles after the procedure was based on the knowledge that follicular development takes an average of 90-120 days from primordial follicle recruitment to antral follicle development, supporting the hypothesis that PRP could stimulate the development of pre-antral follicles and delay atresia.

We demonstrated an improvement in ovarian function in these patients by a decrease in FSH values and an increase in both AMH and the number of antral follicles. These findings are similar to those reported by a recent study of 510 poor responders, in which hormone values and antral follicle counts were compared before and after PRP injection, resulting in a decrease in FSH (20.6 IU/ml *vs*. 16.4 IU/ml; *p*<0.001), and an increase in AMH (0.35 ng/ml *vs*. 0.53 ng/ml; *p*<0.001) and antral follicle count (2.6 *vs*. 4.2; *p*<0.001) (Cakiroglu *et al*., 2022). The same study also evaluated the impact of different variables on the outcomes, and considered 40 years old as a cut-off age for patients who would not benefit from PRP due to failure of ovarian response (sensitivity of 61.54% and specificity of 73.77%). In our study, all patients had an increase in antral follicle count after the procedure; however, this parameter only had a significant impact in patients up to 42 years of age.

A prospective non-randomized controlled trial in which 46 patients with diminished ovarian reserve who underwent PRP injection (study group) *versus* 37 who did not (control group), showed at 3-month follow-up a significant improvement in FSH, AMH and antral follicle count in the study group, while there was no change in the control group (Melo *et al*., 2020). What is interesting about this study, although it was not randomized, is that it had a control group. Similar findings have been reported by other researchers (Sills *et al*., 2018; Sfakianoudis *et al*., 2020; Pacu *et al*., 2021; Panda *et al*., 2020; Petryk & Petryk, 2020).

A recently published study that evaluated the impact on a cohort of 80 women with diminished ovarian reserve or poor responders failed to demonstrate a statistically significant benefit following intra-ovarian PRP. The authors concluded that one of the possible explanations may be due to the inclusion of women with poorer reproductive prognosis, especially in terms of advanced age, and therefore are inclined to infer that the potential effects are still being researched and that these outcomes should be interpreted with caution. So far, they have reported two pregnancies in patients in their 40s with several failed fertility treatments (Barad *et al*., 2022).

Regarding ART outcomes, we demonstrated a significant improvement in the number of retrieved oocytes, number of metaphase II oocytes, number of 2 pronuclei and developing embryos, with respect to the cycle prior to PRP application. There were no differences in fertilization rates. Likewise, Cakiroglu *et al*. (2022) obtained a significant increase in the number of retrieved oocytes (2.2 *vs*. 3.4; *p*<0.001), number of metaphase II oocytes (1.7 *vs*. 2.7; *p*<0.001), fertilization rate (57.6 *vs*. 66.9; *p*<0.008) and number of 2 pronuclei embryos (1.3 *vs*. 2.1; *p*<0.001). Other studies also reported an improvement in assisted fertilization parameters as well as a decrease in cancellation rates (Sfakianoudis *et al*., 2020; Panda *et al*., 2020; Sfakianoudis *et al*., 2019).

The pregnancy rate in our total sample was low. However, it is important to note that if only patients under 40 years of age are included, the pregnancy rate is 27.5% (11/40), compared to 11.36% (5/44) in the older population. According to a retrospective cohort study that analyzed more than 26,000 IVF/ICSI cycles, the cumulative pregnancy rate after a complete IVF cycle was 14.73% for patients included in the Poseidon 3 group, and 6.73% for the Poseidon 4 (Li *et al*., 2019). Our study is still in the follow-up period, and a percentage of patients have cryopreserved embryos that have not yet been transferred, so no results are reported in cumulative pregnancy rate, nor in subsequent ovarian simulations.

Although ART outcomes were better after PRP therapy, we cannot infer an improvement in oocyte quality or demonstrate a real impact of the number of retrieved oocytes and produced embryos on the pregnancy rates. The low number of patients who underwent PGT-A does not allow us to demonstrate a benefit of intraovarian PRP on aneuploidy rates. To date, only one pilot study that included 12 patients has been published, comparing PGT-A results of the cycle following PRP treatment, against those of the previous one. The embryo euploidy rates were 8.11 *vs*. 39.28%, respectively. Although the sample size was very low, they attributed the findings to the local paracrine effect that plasma growth factors may exhibit, correcting meiotic aberrations in human oocytes, directly impacting the rate of euploidy (Merhi *et al*., 2022).

It is unknown what influence the mechanical stimulation produced by ovarian puncture has on the pool of quiescent follicles, and therefore its contribution to the published outcomes. Currently, there is an ongoing prospective randomized study which will compare the results after ovarian PRP with the injection of a platelet-poor plasma fraction (Registration # NCT04278313) (Barad *et al*., 2022).

One of the strengths of this study is its prospective design as well as the unified protocol that we have implemented, with PRP preparation 2 hours before the procedure, performed by the same operator. At the same time, we highlight the comparative design, which allowed the same cohort of patients to be included as their control group, thus monitoring differences in demographic variables.

One of the main limitations is the short follow-up period, as we only evaluated the outcome of the first IVF, ignoring the long-term consequences and the cumulative pregnancy rates. On the other hand, we did not have a control group but each patient was her own control, which is not ideal given the possible regression to the mean in the obtained results. Other limitations were the inclusion of patients with a very poor prognosis and mostly aged, a wide age range, and a high percentage of patients lost to follow-up due to the prospective nature of the study.

Finally, it is important to emphasize that there is a wide heterogeneity on the protocols of this technique, with regard to multiple factors such as: the volume of processed blood, the volume of injected plasma, the method of platelet activation, the plasma injection route, the best timing of the cycle for its application, the number of infusions to be performed, the follow-up time, the time interval until ART, the definition of poor responders. Furthermore, actual evidence is based on a few series of cases, or on prospective controlled and uncontrolled pilot studies, all of which are not randomized. For these reasons, we believe that this technique should be considered experimental and that it is crucial to identify the target patients that could benefit from it, according to different variables.

## CONCLUSION

Intraovarian PRP had a favourable impact on ART outcomes 3 months after injection compared to the previous cycle. There was an improvement in ovarian reserve, with a limited impact on pregnancy rates. Further randomized controlled trials are required to validate our findings.
